# Glycyrrhizin Acid and Glycyrrhetinic Acid Modified Polyethyleneimine for Targeted DNA Delivery to Hepatocellular Carcinoma

**DOI:** 10.3390/ijms20205074

**Published:** 2019-10-12

**Authors:** Mingzhuo Cao, Yong Gao, Mengling Zhan, Nasha Qiu, Ying Piao, Zhuxian Zhou, Youqing Shen

**Affiliations:** 1 Center for Bio-Nanoengineering and Key Laboratory of Biomass Chemical Engineering, Ministry of Education, College of Chemical and Biological Engineering, Zhejiang University, Hangzhou 310027, China; qiunasha@163.com (N.Q.); piaoyingfighting@163.com (Y.P.); zhouzx@zju.edu.cn (Z.Z.); shenyq@zju.edu.cn (Y.S.); 2 Scientific Research and Experimental Center, Henan University of Chinese Medicine, Zhengzhou 450058, China; zml95111@163.com; 3 Henan Province Food and Drug Administration, Food and Drug Evaluation and Inspection Center, Zhengzhou 450018, China; yongg1001@126.com

**Keywords:** gene therapy, glycyrrhizin acid, glycyrrhetinic acid, hepatic tumor targeting, caveolae- and clathrin-independent pathway, HepG2 intraperitoneal tumor model

## Abstract

In the last 2–3 decades, gene therapy represented a promising option for hepatocellular carcinoma (HCC) treatment. However, the design of safe and efficient gene delivery systems is still one of the major challenges that require solutions. In this study, we demonstrate a versatile method for covalent conjugation of glycyrrhizin acid (GL) or glycyrrhetinic acid (GA) to increase the transfection efficiency of Polyethyleneimine (PEI, Mw 1.8K) and improve their targeting abilities of hepatoma carcinoma cells. GA and GL targeting ligands were grafted to PEI via N-acylation, and we systematically investigated their biophysical properties, cytotoxicity, liver targeting and transfection efficiency, and endocytosis pathway trafficking. PEI-GA_0.75_, PEI-GL1_0.62_ and PEI-GL2_0.65_ conjugates caused significant increases in gene transfection efficiency and superior selectivity for HepG2 cells, with all three conjugates showing specific recognition of HepG2 cells by the free GA competition assay. The endocytosis inhibition and intracellular trafficking results indicated that PEI-GA_0.75_ and GL1_0.62_ conjugates behaved similarly to SV40 virus, by proceeding via the caveolae- and clathrin-independent mediated endocytosis pathway and bypassing entry into lysosomes, with an energy independent manner, achieving their high transfection efficiencies. In the HepG2 intraperitoneal tumor model, PEI-GA_0.75_ and PEI-GL1_0.62_ carrying the luciferase reporter gene gained high gene expression, suggesting potential use for in vivo application.

## 1. Introduction

Hepatocellular carcinoma (HCC) is the fifth most common cancer in the world. Unfortunately, most patients are diagnosed at an advanced stage, resulting in higher death rates, and making HCC the second most mortal malignant tumor [1,2]. In addition, there are not many treatment options for late stage HCC due to multiple disadvantages of first-line therapy, such as resistance, lack of target, and severe side effects. In these regards, gene therapy has already been an attractive option to treat HCC. However, what genes should be targeted and how to efficiently deliver therapeutic genes into specific cells and solid tumors are two key issues for gene therapy [3,4]. In recent decades, many non-viral deliveries, especially cationic polymers have attracted extensive attention [5,6,7]. Cationic polymers such as dendrimer polyamide-amine (PAMAM), linear or branched polyethyleneimine (PEI), poly-lysine (PLL), chitosan and their derivatives, have been widely investigated in the gene delivery field owing to their strong stability, low immunogenicity, and controllable structures [8]. The branched PEI25K is considered to be the gold standard in cationic polymer-based gene delivery [9], and is famed for its indiscriminate high transfection efficiency, but also for its severe toxicity [10,11]. However, low molecular weight PEIs (LMW, below 2000 Da) are known for their low toxicity but poor transfection efficiency [12]. The inverse property between transfection efficiency and toxicity of the different weight PEI molecules results in great limitations for their in vivo and in vitro applications.

Many studies have focused on the LMW PEIs’ modifications to improve their transfection efficiency and maintain their low toxicity in vitro and in vivo [12,13]. Hydrophobic modifications, such as ethyl, octyl, octylacrylamide [14,15], dodecyl and benzyl [16], lipoic acid and cholic acid [17], are one of the most effective strategies. Despite the large differences in grafting substituents, the performance of many modified PEIs was greatly enhanced in gene expression activity compared to unmodified PEIs. Even some modified PEIs had comparable or much higher efficacy than the 25 K PEI [17]. Some studies also reported that hydrophobically modified LMW PEIs caused an increase in cytotoxicity compared to parent PEIs; however, this was much lower than the 25 K PEI [14,18]. Therefore, the hydrophobicity and substituting degrees of conjugated groups are of much significance, can have important influences on the binding and releasing ability of DNA, toxicity and transfection efficiency.

A suitable targeting ligand for drug delivery is determined by the specificity required, the cost of the ligand, and market supply [19,20]. In many previous studies, cationic polymers have been functionalized with targeting ligands including mannose [21], folic acid, galactose [22], and polypeptide [23], which allowed the functionalized polymers to bind cell-specific receptors and trigger receptor-mediated endocytosis. Glycyrrhizin acid (GL) and glycyrrhetinic acid (GA, an active metabolite of GL) are pentacyclic triterpenoid compounds, that are mainly isolated from licorice, the root of *Glycyrrhiza glabra* L. They are chemically similar to the aldosterone steroid ring, can reduce non-specific liver inflammation, and have been used for the treatment of hepatic fibrosis and hepatitis in Asia for more than 30 years [24]. Many related studies [25,26] have confirmed the existence of a large number of glycyrrhetinic acid-specific binding sites on the membrane of the liver parenchyma, and many studies have also reported that GA or GL modified vectors can specifically bind to GA receptors, exhibiting favorable liver targeting properties [27,28,29,30,31].

Herein, we hypothesized that glycyrrhetinic acid and glycyrrhizin acid modified LMW PEIs could acquire high transfection efficiency and liver targeting. In this study, GL and GA modified PEI conjugates were synthesized by one step or two steps N-acylation. GA was grafted to PEI at three different substitution amounts. We found that gene expression of PEI-GA conjugates was optimal at feed ratio 1 (GA: PEI), accordingly PEI–GL conjugates only fixed at a feed ratio of 1. Biophysical characterization, cytotoxicity, and pDNA delivery capability of all modified PEIs were evaluated. To verify liver targeting ability of modified PEIs, the effect of free GA on their transfection efficiency in HepG2 and SW480 cells was also investigated. Moreover, to elucidate the receptor-mediated endocytosis mechanism of the conjugates in vitro, we investigated their nucleus traffic by flow cytometry and laser confocal microscopy. A HepG2 hepatoma intraperitoneal model was constructed to verify their gene expression in vivo.

## 2. Results and Discussion

### 2.1. Synthesis and Characterization of GA or GL Functionalized PEIs

As described above, LMW PEIs, have been the most widely studied vectors for non-viral gene delivery due to their low toxicity and easy modification process. Hydrophobic modification has been widely demonstrated as a very effective method to increase the transfection efficiency of low molecular weight PEIs. In this study, we aimed to synthesize two hydrophobic targeting ligands modified PEIs for targeted DNA delivery to hepatocellular carcinoma. Varied amounts of GA were substituted to PEI 1.8 K. As for GL modification, they were synthesized in two steps as shown in Scheme S1B. Since GL has three active carboxyl groups, twofold ethylamine (1) or histamine (2), these were firstly reacted with GL to prevent cross-linking when GL directly reacted with PEI, and as previously reported [30]. The successful conjugation of GA or GL to the PEI polymer was confirmed by the 1HNMR, IR and UV absorption.

**Figure 1:**
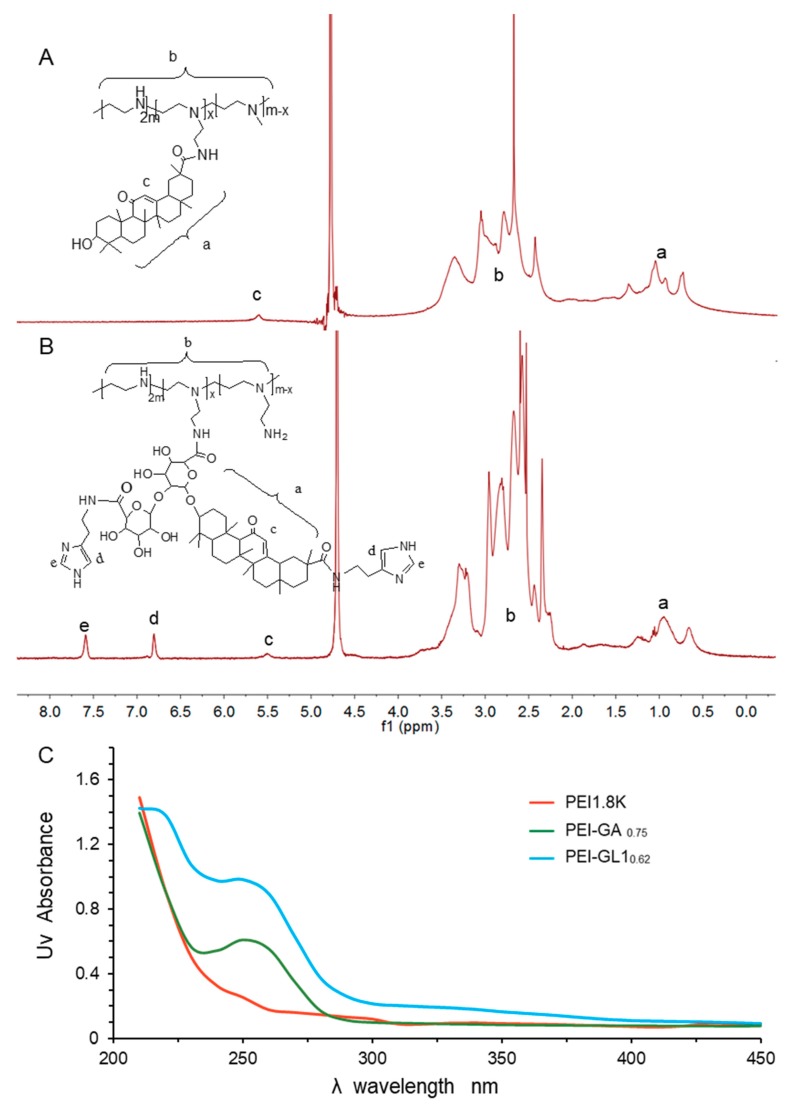
Characterization of and glycyrrhetinic acid (GA) or glycyrrhizin acid (GL) modified PEIs. (**A**) 1H-NMR of polyethyleneimine (PEI)-GA_1.7_, (**B**) PEI-GL2_0.65_. (**C**) UV absorbance of PEI-GA_0.75_ (0.1 mg/mL), PEI-GL1_0.62_ (0.15 mg/mL) and PEI1.8K (0.384 mg/mL).

As shown in Figure 1A,B, 1H-NMR (400MHz, D_2_O, δ): 3.4−2.0 ppm (b, −NHCH_2_CH_2_−), and at 5.5 ppm and 2.0−0.43 ppm (a, c, pentacyclic triterpenoid, GA, GL), at 7.6ppm and 6.7ppm (histamine, PEI-GL2, Figure 1B); moreover, FT-IR peak of −CO-NH− at 1649.5 or 1652.13 cm^−1^ (Figure S1) in PEI-GA or PEI-GL further indicated the successful synthesis of PEI-GA and PEI-GL. From the UV absorption of PEI-GA and PEI-GL conjugates at wavelength of 250 nm, the degree of GA substituting was 0.75, 1.1 and 1.7, and the degree of GL (1, 2) substituting was 0.62 and 0.65 respectively (see Table 1) (the conjugate PEI-GA0.75 represents a 1.8K PEI conjugated with an average number of 0.75 GA molecules. For conjugates PEI-GL10.62 and PEI-GL20.65, they represent a 1.8K PEI conjugated with an average number of 0.62 GL1 molecules or 0.65 GL2 molecules. Moreover 1 or 2 represents a GL molecule coupled with 2 ethylamine molecules or 2 histamine molecules). All the five lyophilized GA-PEI or GL-PEI conjugates were white crystalline powders with good water solubility.

### 2.2. Biophysical Characterization of GA or GL Modified PEIs

**Figure 2:**
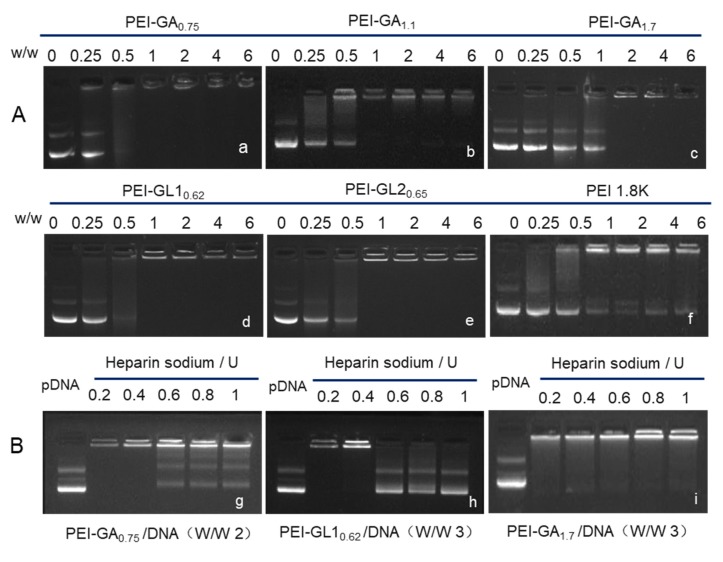
Agarose gel assay. (**A**) pDNA binding ability assay, PEI-GA (GL)/pLUCI polyplexes were prepared at various weight ratios (W/W) from 0.25 to 6 with fixed amount of pDNA (0.4 μg, pGL4.13 luciferase plasmid) in pH 7.4 HEPEs solution. (**B**) Heparin competitive binding assay. 0.2–1 U heparin sodium were added in newly prepared PEI-GA_0.75_/pDNA (W/W 2), PEI-GL1_0.62_/pDNA (W/W 3) and PEI-GA_1.7_/pDNA (W/W 3) polyplexes, and then incubated for 30 min at room temperature.

We assumed that the added hydrophobicity would allow PEI to condense DNA better. As shown in Figure 2A, the gel electrophoresis results indicated that the amount of GA substitution played a significant role in pDNA condensing capacity. PEI-GA_0.75_ and PEI-GL1_0.62_ had better DNA binding ability, they retarded the migration of pDNA at the weight ratio of 0.5. Meanwhile, the PEI-GA_1.7_ conjugate with high substitution, completely retarded pDNA at the weight ratio of 2. The PEI-GA_1.1_ and PEI-GL2_0.65_ conjugates could completely inhibit pDNA migration at weight ratio of 1. The results were consistent with previously published conclusions [17,32]. When the substitution of hydrophobic molecules on 1.8K PEI increased, the capacity of binding pDNA gradually decreased. PEI-GA_1.7_ had a weaker pDNA binding capacity, probably due to the excess of hydrophobic attractions among GA which drive GA to the core of the polyplex [10,32,33,34].

Upon entering the cells, the release of pDNA from the polyplex is also important for an efficient gene transfection. The heparin competitive binding assay was used to evaluate the binding affinity between conjugates and pDNA. As shown in Figure 2B, pDNA could release from the three polyplexes by adding 0.6 U of heparin sodium, and only PEI-GL1_0.62_ had achieved complete dissociation under the competition with 0.6 U of heparin sodium. However, PEI-GA_0.75_/pDNA polyplexes could not completely release pDNA even when heparin was increased to 1U, when PEI-GA_1.7_/pDNA polyplex could hardly release pDNA. An appropriate binding affinity between conjugates and pDNA was critical in high transfection efficiency [14]. The two glucopyranoside of GL may have played a critical role in releasing the pDNA.

**Figure 3:**
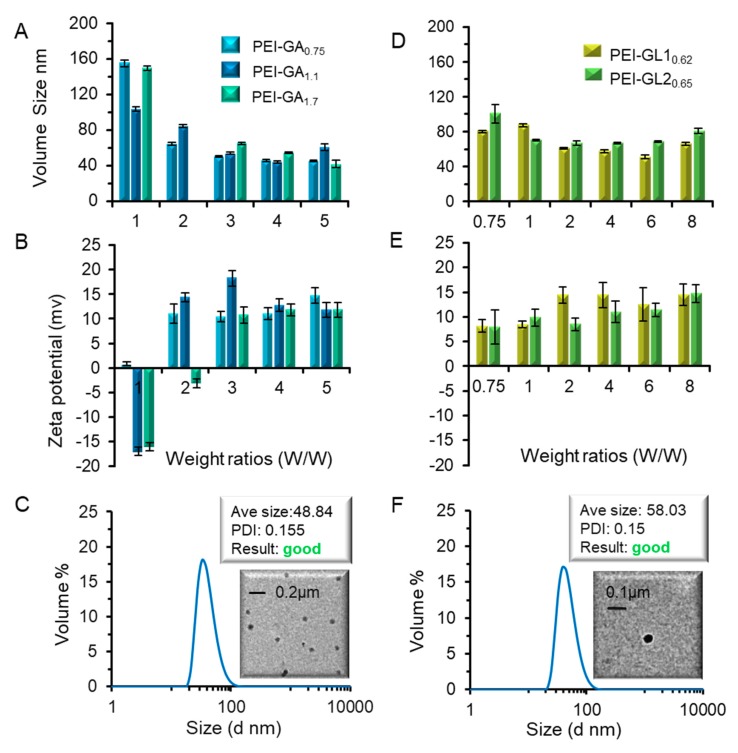
Sizes and zeta potentials of PEI-GA and PEI-GL polyplexes. (**A**,**D**) Sizes and (**B**,**E**) Zeta potentials of PEI-GA (**A**,**B**) and PEI-GL (**D**,**E**) polyplexes at various weight ratios. (**C**,**F**) Volume size distribution of PEI-GA_0.75_/pLUCI and PEI-GL1_0.62_/pLUCI at weight ratio of 3, and their morphologies observed by TEM after staining with phosphato-tungstic acid (PTA). Scale bar 100 nm.

It has been confirmed that nanoparticles size would greatly influence their cellular uptake, and small particles size with ∼100 nm are more favorable for internalization [35]. As shown in Figure 3, all the conjugates can condense pDNA into small nanoparticles with sizes ranging from 46–66 nm at higher weight ratios, that are much smaller than unmodified PEI1.8K [36]. Interestingly, the sizes of PEI-GL polyplexes were significantly smaller than the PEI-GA_0.75_ polyplex at the weight ratio of 1. While at the weight ratio of 3, the sizes of PEI-GL polyplex were a little larger than that of PEI-GA_0.75_/pDNA, this was further confirmed by transmission electron microscopy (TEM) images, as shown in Figure 3C,F. The substitution degree of GA had a minimal effect on the sizes of PEI-GA/pDNA polyplexes at high weight ratios. We found that the GA substitution degree played an important role in polyplexes charge conversion. Even at the weight ratio of 2, the surface charge of PEI-GA_1.7_/pDNA polyplexes was negative, which was concordant with the previous DNA binding assay. For PEI-GL1_0.62_ and PEI-GL2_0.65_ conjugates, the zeta potentials remained positive at a low weight ratio of 0.75, which are much higher than all the PEI-GA conjugates. In brief, the zeta potentials of all GA or GL modified conjugates were equivalent to that of 1.8K PEI polyplexes at high weight ratios, and which are much lower than many widely used cationic polymers [37] or cationic liposomes [38], which may explain their low cytotoxicity.

### 2.3. Cytotoxicity and Gene Transfection in Vitro

**Figure 4:**
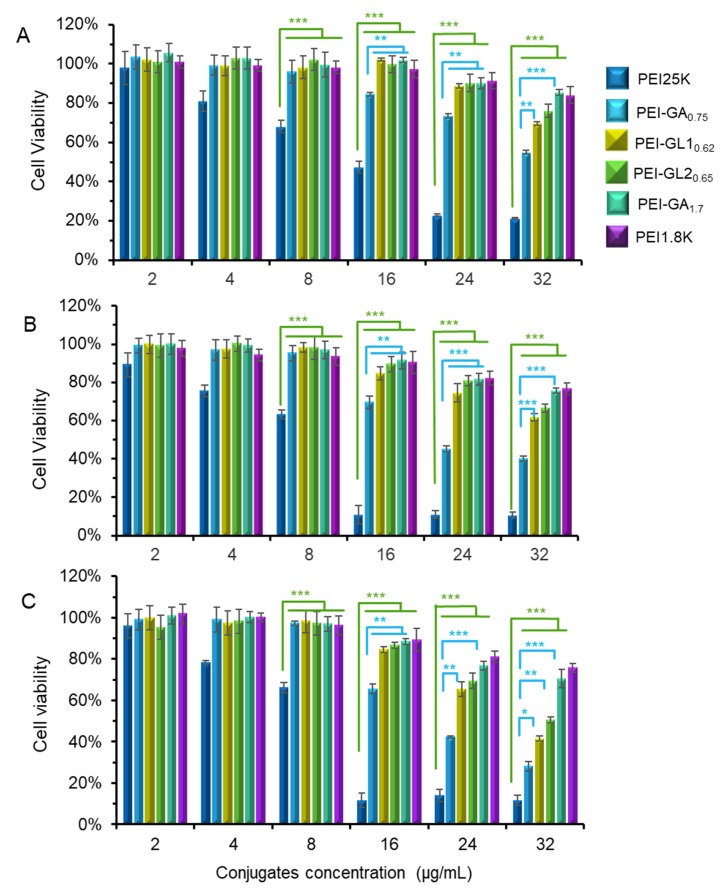
Cell viability of the PEI-GA and PEI-GL conjugates at concentrations ranging from 2 to 32 μg/mL after 48h incubation, using PEI1.8K and PEI25K as controls. *** *p* value < 0.001. ** *p* value < 0.01 using *t*-test (unpaired). Results are shown as mean ± SD (*n* = 3). (**A**) HepG2 cells, (**B**) SW480 cells, and (**C**) A549 cells.

The cytotoxicity of PEI-GA and PEI-GL conjugates were investigated on HepG2, SW480 and A549 cell lines in the range of 2.0–32.0 μg/mL after 48 h incubation. Compared to 1.8K PEI, when the concentration was higher than 16 μg/mL, the PEI-GA_0.75_ conjugates revealed a slightly elevated cytotoxicity as expected in all three cell lines and owing to the surface hydrophobic modification [16,17,34,39]. However, when the concentration was higher than 4μg/mL, they all exhibited much lower cytotoxicity than PEI 25K (*p* < 0.01 or *p* < 0.001). For GA modified PEIs, as shown in Figure 4A–C, the higher the amount of GA grafted to PEI, the lower the cytotoxicity was obtained. Interestingly, the PEI-GL1_0.62_, which produced the highest luciferase transfection efficiency, was found to have lower cytotoxicity than the PEI-GA_0.75_ and at all tested concentrations.

**Figure 5:**
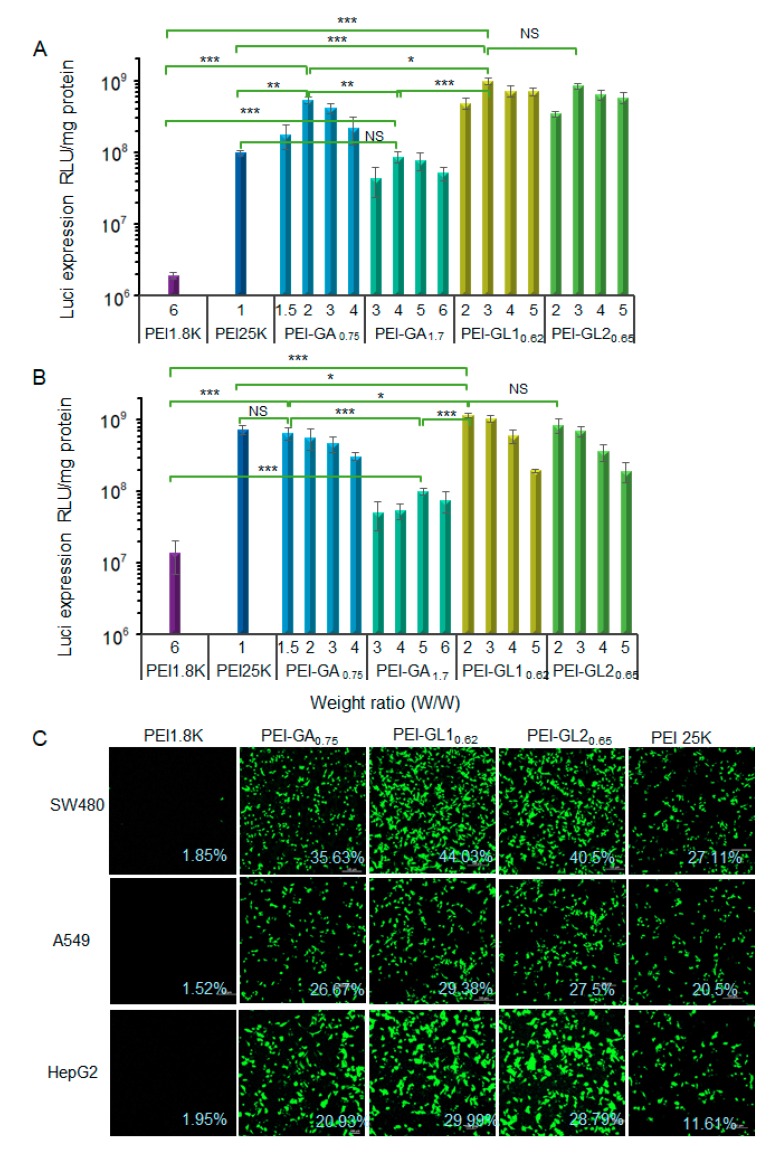
In vitro gene transfection efficiency. Luciferase expression efficiency of PEI-GA/pLUCI and PEI-GL/pLUCI polyplexes and the controls (PEI.8K and PEI25K) in serum-free medium on HepG2 (**A**) and SW480 (**B**) cancer cells with 2.25 μg/mL Luciferase plasmid concentration. Results are shown as mean ± SD (*n* = 3). Statistically significant differences are indicated by * *p* < 0.05, ** *p* < 0.01 and *** *p* < 0.001 using *t*-test (unpaired). (**C**) Expressing green fluorescence protein (EGFP) expression efficiency of the three polyplexes and the controls on SW480, A549 and HepG2 cells with 2.5 μg/mL EGFP plasmid concentration observed by confocal laser scanning microscope (CLSM). All scale bars represent 100 μm and GFP-positive cells are measured by flow cytometry.

**Figure 6:**
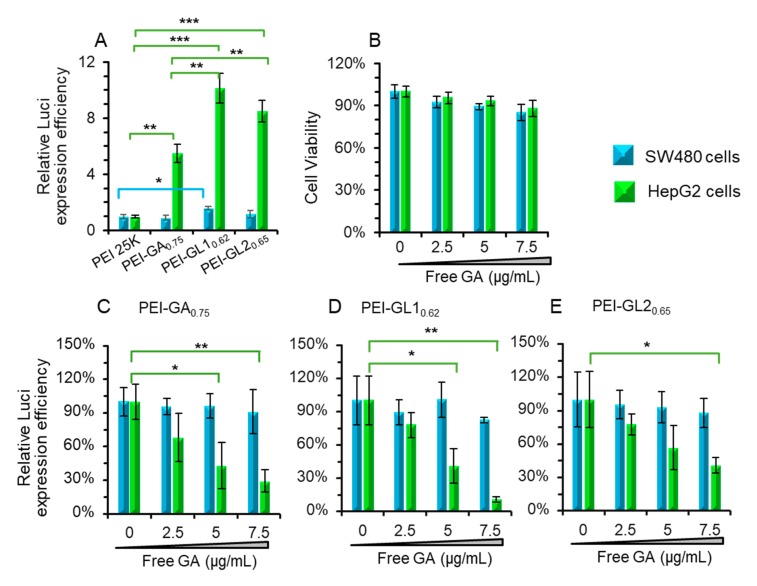
Free GA competition assay. (**A**) Relative transfection efficiency of PEI25K, PEI-GA and PEI-GL conjugates at their optimum weight ratio in SW480 and HepG2 cells. Values are given as the mean ± SD of triplicates. Statistically significant differences (**A**) are indicated by ** *p* < 0.01 and *** *p* < 0.001 using t-test (unpaired). (**B**) Cell viability of free GA in SW480 and HepG2 cells. Effect of free GA (0, 2.5, 5 and 7.5 μg/mL) in relative Luci expression efficiency of PEI-GA_0.75_/pLUCI (**C**), PEI-GL1_0.62_/pLUCI (**D**) and GL2_0.65_/pLUCI (**E**) on HepG2 and SW480 cells. The results are shown as mean ± SD (*n* = 3). Statistically significant differences (**C**–**E**) are indicated by * *p* < 0.05 and ** *p* < 0.01 using *t*-test (unpaired).

The in vitro transfection efficiency of PEI-GA or PEI-GL conjugates at various weight ratios was also evaluated on the three cell lines using pGL3 (Figure 5A,B) and expressing green fluorescence protein (EGFP) (Figure 5C) as reporter genes. In comparison to 1.8K PEI, a significant increase in gene expression of 513.8- and 431.3-fold was observed in HepG2 cells cultured in serum-free medium, and for the two GL modified PEIs under their optimum weight ratio. Furthermore, the transfection efficiency of PEI-GA_0.75_ and PEI-GL1_0.62_ polyplexes in HepG2 cells was 5.5 times and 10.2 times higher than that of the PEI 25 K polyplex at their optimal weight ratios, but only equal to or a bit higher than that of PEI 25 K polyplex in SW480 cells (Figure 6A), and slightly lower than or equal to that of PEI 25 K polyplex on A549 cells (Figure S2). Both PEI-GA_0.75_ and PEI-GL1_0.62_ polyplexes showed high selectivity to HepG2 cells (shown in Figure 6A), which overexpressed GA receptor [33,40,41]. Overall, transfection efficiency of GL modified PEIs was much higher than GA modified PEIs polyplexes in three cell lines (*p* < 0.01, 0.05). As for PEI25K, the presence of 10% FBS resulted in a significant reduction in gene expression (*p* < 0.001) (Figure S3A–C), and similar results were observed in all modified PEIs polyplexes, while the inhibition was much attenuated compared to PEI 25 K. PEI-GL1_0.62_/pLUCI at the weight ratio of 4 produced the highest gene expression in HepG2 cells, which was 76.8-fold higher than that of the 1.8K PEI/pLUCI, and 5.8-fold higher than that of the 25 K PEI/pLUCI.

The results of EGFP (Figure 5C and Figure S3) and TRAIL transfection (Figure S4) were coincident with the results of pGL3 transfection. The PEI-GL1_0.62_/pEGFP polyplex achieved the highest transfection efficiency in SW480, HepG2 and A549 cells cultured in serum-free medium. The positive percentage of transfected cells were 44.33%, 29.99% and 29.38%, compared with 27.11%, 11.61% and 20.5% for PEI 25K/EGFP polyplex transfected cells. In the containing 10% serum medium (Figure S1C), the EGFP expression efficiency for PEI-GL1_0.62_/EGFP polyplex at the weight ratio of 6 on SW480 and HepG2 cells were 19.38% and 17.91% respectively, while the PEI 25K/pEGFP polyplex only transfected 3.71% and 2.26% of cells. As shown in Figure S4, PEI-GL1_0.62_/pTRAIL expressed TRAIL more efficiently than the PEI/pTRAIL polyplex and thus induced more efficient apoptosis in the cells. Thus, the PEI-GL1_0.62_ conjugate is better for gene delivery vectors compared to the PEI 1.8 K, PEI 25 K and PEI-GA.

In some previous studies [16,32,42], it had been reported that a hydrophilic/hydrophobic balance must be achieved in the hydrophobic modification to achieve optimal gene transfection efficiency. GA is a water-insoluble molecule, and its solubility is only 6.32 mg·L^−1^ in water at 37 °C. However, GL, containing a glycoside with a molecular weight of about 400, is easily soluble in hot water, but insoluble in acetone. In addition to evaluating the effects of different substituting degrees of glycyrrhetinic acid on pDNA binding capacity and transfection efficiency, we also evaluated the effect of GA and GL molecules with different hydrophobic properties on the improvement of transfection efficiency. Most of the results are consistent with the previously reported results, but there are some slightly different results. The medium-substituted PEI-GA_1.1_ conjugate had an intermediate pDNA binding capacity, but it gained the worst transfection efficiency on all three cell lines (Figure S5). Although the PEI-GL1_0.62_/pDNA and PEI-GA_0.75_/pDNA polyplexes did not significantly differ in the binding ability (Figure 2A), particle size and zeta potential (Figure 3), the PEI-GL1_0.62_ conjugate achieved a better transfection efficiency (Figure 5) in vitro due to its appropriate binding affinity with pDNA.

### 2.4. Effect of free GA on the Transfection Efficiency

As shown in Figure 5 and Figure S3, the three conjugates (PEI-GA_0.75_, PEI-GL1_0.62_ and PEI-GL2_0.65_) achieved a significantly increased gene expression efficiency in all three cell lines, but their multiples were different and increased compared to the 25 K PEI (gold standard) in transfection efficiency (shown in Figure 6A). To further verify their good selectivity for HepG2 cells, we performed a free GA competitive assay in HepG2 and SW480 (GA-R negative) cells. As shown in Figure 6C–E, the free GA presented a dose dependent inhibition on luciferase expressing efficiency of the three polyplexes, especially with 7.5 μg/mL free GA pretreatment (no obvious cytotoxicity, as shown in Figure 6B). The transfection efficiency of the PEI-GA_0.75_/pLUCI, PEI-GL1_0.62_/pLUCI and PEI-GL2_0.65_/pLUCI polyplexes in HepG2 cells was significantly reduced by 70.6%, 89.2%, and 59.2%, respectively. However, the free GA had little effect on the transfection efficiency in SW480 cells.

The transfection efficiency of the PEI-GL2_0.65_/pLUCI polyplex was a little lower than that of the PEI-GL1_0.62_/pLUCI polyplex, and the inhibition on transfection efficiency of PEI-GL2_0.65_/pLUCI polyplex was more gentle than that of the PEI-GL1_0.62_/pLUCI polyplex, which was probably due to the sterically hindered effect of histamine moiety coupled to GL. Furthermore, the PEI-GL1_0.62_ showed a slightly better performance in respect of specificity to the GA receptor compared to the PEI-GA_0.75_. In combination with the above transfection results, it could be inferred that the glucoside of GL played two roles, one was to affect the hydrophilic/hydrophobic balance to achieve optimal gene transfection efficiency (Figure 5), and the other was to act as a linger to render PEI-GL1_0.62_ conjugate to produce a more efficiently interactive with GA receptors (Figure 6). These results strongly confirmed that the GA receptor on the surface of HepG2 cells played an important role in the PEI-GA_0.75_, GL1_0.62_ and GL2_0.65_-mediated gene delivery.

In recent decades, several types of receptor-mediated liver-targeting gene delivery systems have been developed for the treatment of HCC, including hyaluronic acid receptor [43], folate receptor [44], sialic acid glycoprotein receptor [45,46], transferrin receptor [47] and glycyrrhetinic acid receptor (GA-R) [1,48], etc. GA-R has been considered as an up-and-coming target due to its excellent targeting ability for HCC. In this study, we designed two kinds of non-virus gene vectors for a liver targeting gene delivery system that is based on the GA receptor. Due to hydrophobic modification of the GA or GL molecules, PEI-GA_0.75_, PEI-GL1_0.62_ and PEI-GL2_0.65_ conjugates achieved significant enhancements in gene transfection efficiency and for a good selectivity towards hepatoma cells.

### 2.5. Endocytic Pathway and Intracellular Trafficking

It was reported that targeting ligands, such as folic acid and transferrin, could influence the intracellular processing of the gene delivery vehicle [49]. The Free GA competitive assay results indicated that GA modified PEI has successfully produced specific cellular interactions. To illustrate the effects of GA and GL targeting ligand on the intracellular trafficking, the endocytic pathways of PEI-GA_0.75_/pLUCI, PEI-GL1_0.62_/pLUCI and PEI-GA_1.71_/pLUCI polyplexes at their optimum weight ratios (2, 3 or 3, respectively) were investigated. To preclude the effect of inhibitors’ nonspecific toxicity on the transfection activity, all the HepG2 cells retained more than 85% following exposure to these inhibitors and at their working concentrations (data not shown).

**Figure 7:**
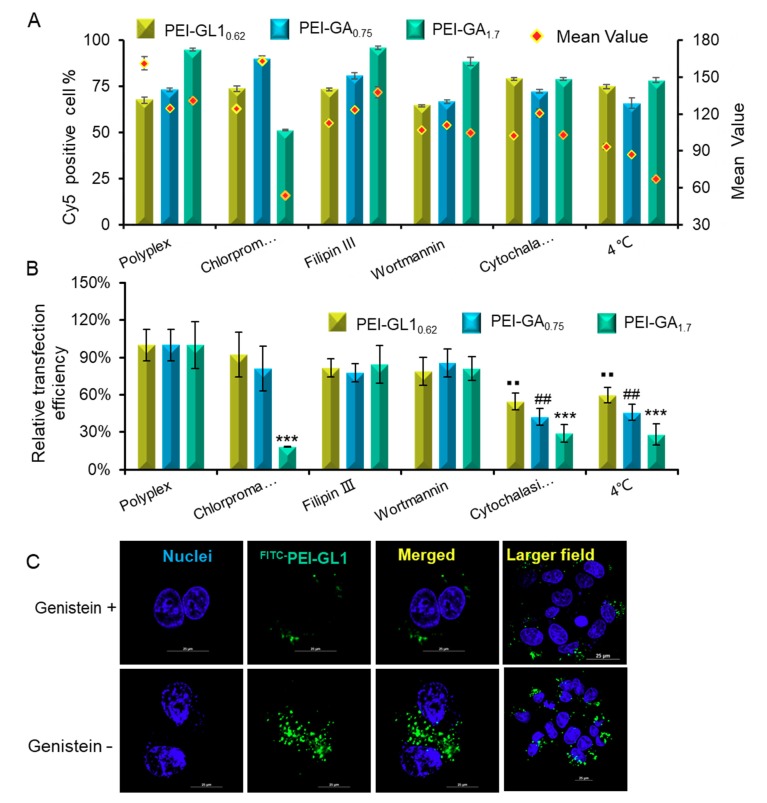
Endocytosis inhibitor effects on cellular uptake (**A**,**C**) and transfection efficiency (**B**). (**A**) Cy5-positive HepG2 cells measured by flow cytometry after pretreatment of the cells with four inhibitors (Chlorpromazine, Filipin Ⅲ, Cytochalasin D and Wortmannin) for 0.5 h and then cultured with the three polyplexes for 2 h in serum-free medium at 37 °C or 4 °C. (**B**) The effect of the four inhibitors on transfection efficiency of the three polyplexes in HepG2 cell after 48 h at 37 °C or 4 °C. The results are shown as mean ± SD (*n* = 3). Statistically significant differences are indicated by ** (^##^, ▪▪) *p* < 0.01 and *** *p* < 0.001 using t-test, compared to polyplex. (**C**) The effect of Genistein on cell phagocytosis observed by confocal laser scanning microscopy after 2 h post-transfection. The cell nuclei were stained with Hoechst 33342 (blue). Polyplexes labeled with FITC (See Supporting Information for details) were shown in green. All scale bars represent 25 μm.

As expected, all inhibitors resulted in similar uptake inhibition effects to PEI-GA_0.75_/pLUCI and PEI-GL1_0.62_/pLUCI polyplexes, while also slightly different to PEI-GA_1.71_/pLUCI polyplex. As shown in Figure 7A, the flow cytometry results showed that chlorpromazine, wortmannin, filipin III, or cytochalasin D did not have any noticeable effects on the cellular uptake of the former two polyplexes, However the chlorpromazine suppressed the cellular uptake of the PEI-GA_1.71_ polyplex. The incubation at 4 °C caused a slight inhibitory effect of the three polyplexes. The fluorescence mean value of the three polyplexes all decreased, with the PEI-GA_0.75_/pLUCI and PEI-GL1_0.62_/pLUCI polyplexes decreasing by 33%, and PEI-GA_1.71_ by 50%.

**Scheme 1:**
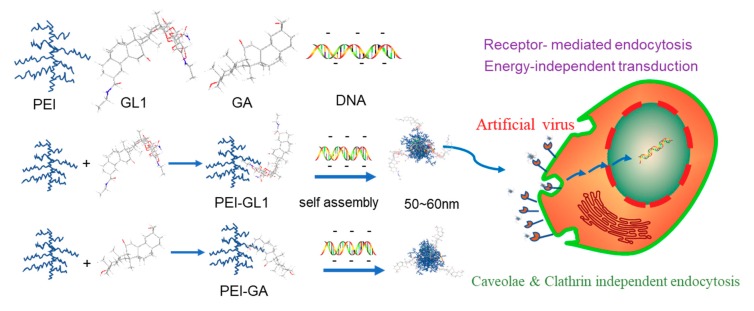
Transfection mechanisms of GA and GL modified PEIs.

The luminometer results (Figure 7B) were consistent with the flow cytometry results. Filipin Ⅲ and wortmannin treatments caused a small decrease in transfection efficiency of the three polyplexes. It could be indicated that the three polyplexes did not delivered pDNA into HepG2 cells not mainly via the micropinocytosis and caveolae pathways [49,50]. Cytochalasin D, inhibitors of caveolae, clathrin and micropinocytosis, inhibited the transfection efficiency of the three polyplexes approximately with 45.1% (*p* < 0.01), 57.3% (*p* < 0.01) and 70.8% (*p* < 0.001), respectively. Chlorpromazine strongly inhibited the transfection efficiency of the PEI-GA_1.712_/pLUCI polyplex (81.5%, *p* < 0.001); however, it had a slight inhibitory effect on the PEI-GA_0.75_/pLUCI and PEI-GL1_0.62_/pLUCI polyplexes. The above results indicated that PEI-GA_1.71_/pLUCI polyplex was mainly endocytosed via a clathrin-mediated pathway [19]. Genistein is a tyrosine kinase inhibitor, known to disrupt caveolae-dependent endocytosis and caveolae- and clathrin-independent endocytosis [51], which resulted in a 30.8% and 64.1% decrease in intracellular uptakes of the PEI-GA_0.75_/pLUCI and PEI-GL1_0.62_/pLUCI polyplexes (Figure S6, Figure 7C). Therefore, we could deduce that the endocytosis of the PEI-GA_0.75_/pLUCI and PEI-GL1_0.62_/pLUCI polyplexes was mainly performed via a caveolae- and clathrin-independent pathway, similar to that of the SV40 virus [52,53]. In addition, this endocytosis pathway was a dynamin Ⅱ independent process [54], consistent with the above-mentioned uptake and transfection results at 4 °C. However, the luciferase expression of the two polyplexes at 4 °C decreased by 54% and 40% (*p* < 0.01), respectively. This energy-independent transduction of PEI-GL1_0.62_ and PEI-GA_0.75_ conjugates might be closely correlated with the GA receptor expressed on the surface of HepG2 cells. In general, the GA receptors mediated endocytosis of the PEI-GA_0.75_/pLUCI and PEI-GL1_0.62_/pLUCI polyplexes may have played an important role in their relatively high transfection efficiency on HepG2 cells. The detailed endocytic process of the PEI-GA_0.75_/pLUCI and PEI-GL1_0.62_/pLUCI polyplexes is shown in Scheme 1.

**Figure 8:**
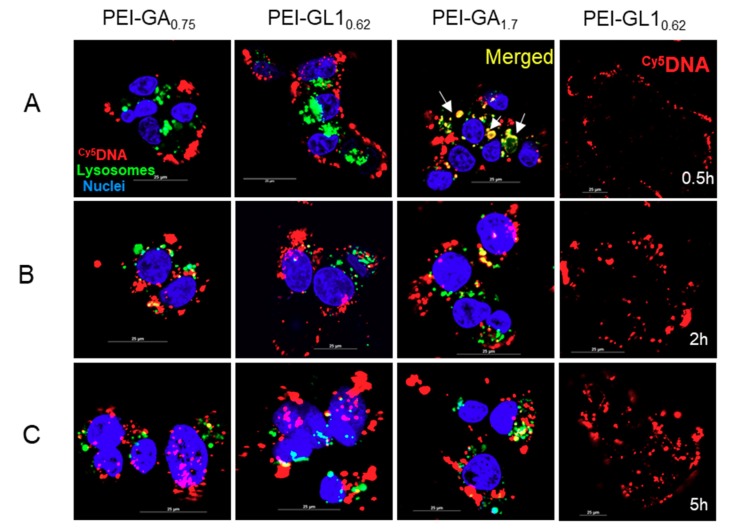
Confocal images of the three polyplexes. PEI-GL1_0.62_/pLUCI, PEI-GA_0.75_/pLUCI and PEI-GA_1.7_/pLUCI cultured for 0.5 h (**A**), 2 h (**B**) and 5 h (**C**) on HepG2 cells. The images were taken from Cy5-DNA (red), lysotracker green (green), Hoechst 33342 (blue) channels, ^Cy5^DNA dose was 0.35 μg/mL. All scale bars represent 25 μm.

Subsequent intracellular localization studies further confirmed the results of the endocytosis inhibitors. The intracellular fate of the three polyplexes was observed by confocal laser scanning microscope (CLSM) using Cy5 -labeled DNA (red) in HepG2 cells. As shown in Figure 8, after 0.5 h of transfection, PEI-GA_0.75_/^cy5^pLUCI and GL1_0.62_/^cy5^pLUCI polyplexes attached to the cell membrane. However, the red fluorescence of the DNA did not overlap with the green fluorescence of lysosomes. In contrast, many overlapping yellow dots of the red fluorescence of DNA and lysosomes green fluorescence were observed with the PEI-GA_1.7_/^cy5-^pLUCI polyplex. The result was consistent with similar findings that were reported by Sun Y et al. and showed that the mannose ligand facilitated cellular uptake of man-PEI–TEG polyplexes via an alternative pathway involving receptor-mediated endocytosis [55]. The PEI-GA_0.75_/^cy5^pLUCI and GL1_0.62_/^cy5^pLUCI polyplexes were probably endocytosed via the GA receptor and thus bypassed the lysosomes, while the PEI-GA_1.7_/^cy5-^pLUCI polyplex was endocytosed via clathrin and trafficked by lysosomes.

Notably, 2 h after transfection (Figure 8b), and the fluorescence of ^cy5-^DNA was still observed on the cell membrane with much stronger signal. Most of the red DNA fluorescence still did not overlapped with the lysosomes’ green fluorescence, and some of the red DNA fluorescence co-located into cell nuclei and as shown in a 2 h video for the PEI-GA_0.75_/pDNA (Supplement Materials Video S1). As shown in Figure 8C, most of the nuclei localizations of the PEI-GA_0.75_/^cy5^pLUCI and GL1_0.62_/^cy5^pLUCI polyplexes were observed within 5 h, despite not being as rapid as some quaternary ammonium cationic vectors [56,57] in our laboratory. Meanwhile, for the PEI-GA_1.7_/pLUCI polyplex with lower transfection efficiency, almost no DNA red fluorescence was observed in the nucleus, suggesting that its rate-limiting steps during transfection is probably due to intracellular trafficking. It was probable that the GA molecule was at the surface of the polyplex, and this had been confirmed by other studies [32] and was consistent with the results of the above gel electrophoresis studies. Compared with the PEI-GA_0.75_/pLUCI polyplex, more pink dots of the PEI-GL1_0.62_/pLUCI polyplex were observed at 2 h and 5 h post transfection, which was consistent with the above-mentioned transfection and free GA competitive results.

Gene therapy is a promising and attractive option for treating hard-to-cure diseases such as HCC, and it has achieved some breakthrough results. In 2017, the FDA approved Tisagenlecleucel as the first gene therapy for the treatment of acute lymphoblastic leukemia for patients under 25 years of age. However, the key issue with gene therapy is the method of the gene drug delivery to the target cells, because the nucleic acid drug must overcome a series of biological barriers such as distribution in the body, cell uptake through endocytosis, intracellular transport, pDNA release, and nucleus entry, etc. Therefore, the cell uptake mechanism and subsequent endocytic processing are important design parameters for gene delivery vectors. Some previous studies reported that targeting ligand modified polymers and nanomaterials may result in a complex trafficking behavior [51]. A similar study reported that the PEI–Fol (1.1) (with 1.1 folate moieties per polymer chain, 25 K PEI) yielded gene expression levels that were roughly five-fold greater than the unmodified PEI since PEI-Fol(1.1)/pDNA polyplexes was predominantly endocytosed via a caveolar process rather than a nonspecific combination of both caveolin-1 and the clathrin heavy chain for unmodified PEI [49]. As shown in Scheme 1, the PEI-GA_0.75_ and PEI-GL1_0.62_ conjugates, just like SV40 virus, both endocytosed via the caveolae- and clathrin-independent pathway and in an energy-independent manner. This was the key factor for their high transfection efficiency.

### 2.6. In Vivo Gene Transfection

**Figure 9:**
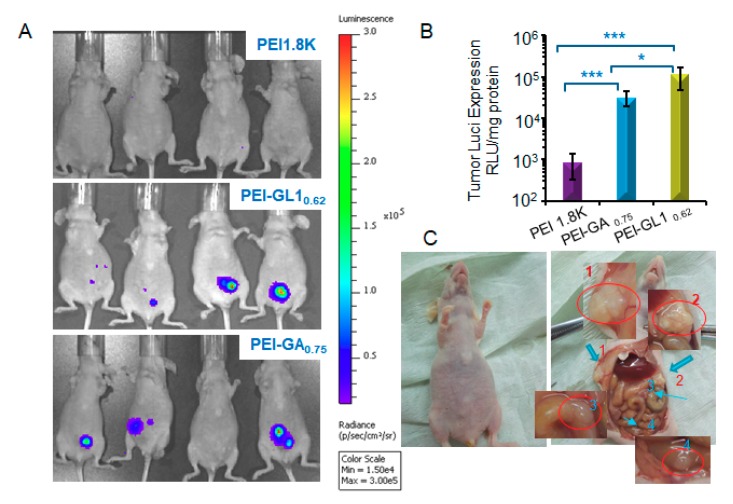
In vivo Luciferase expression efficiency of the polyplexes and the PEI1.8K control. (**A**) Tumor bioluminescent image and (**B**) the corresponding chemiluminescence measurements of the dissected tumor tissues in the HepG2- intraperitoneal tumor nude mice model at 48 h post i.p injection of PEI/pLUCI, PEI-GA_0.75_/pLUCI and PEI-GL1_0.62_/pLUCI polyplexes. The luciferase plasmid dose was 0.5 mg·kg^−1^; 48 h in vivo transfection, *n* = 4 mice in each group. Statistically significant differences are indicated by * *p* < 0.05 and *** *p* < 0.001 using *t*-test. (**C**) Tumor nodules of representative mice. Arrows show tumors around the liver, and intestines, or in the intestines.

In vivo transfection of PEI1.8K/pLUCI, PEI-GA_0.75_/pLUCI and PEI-GL1_0.62_/pLUCI was evaluated in the HepG2 intraperitoneal tumor model to mimic the late stage of hepatic metastases. Twenty days after inoculation, the tumor-bearing mice were administered with the above three polyplexes at a dose of 0.5 mg kg^−1^ of pLUCI by i.p. injection. After 48 h of transfection, all the PEI-GL1_0.62_/pLUCI polyplex treated mice with showed chemiluminescence, and three-quarters of the PEI-GA_0.75_/pLUCI polyplex injected mice presented chemiluminescence, whereas none of the mice treated with PEI/pLUCI polyplex had detectable chemiluminescence (Figure 9A). As shown in Figure 9B, the quantitated luciferase expression in homogenized tumors that were transfected by the PEI-GL1_0.62_/pLUCI polyplex was 57 times higher than that of those transfected by PEI/pLUCI, and 3.5 times higher than that of those transfected by the PEI-GA_0.75_/pLUCI polyplex. These results were consistent with the transfection results in medium containing 10% FBS (shown in Figure S3). Besides, just like classical subcutaneous tumor, small volume tumors may achieve better transfection results (Figure 9C). In addition, the luciferase expression was very low in all the mice’s livers and were almost undetectable (Figure 9A).

The clinical application of local–regional treatment in liver, ovarian and pancreatic cancers has been gradually increasing [58,59]. In this study, we constructed a murine model of peritoneal hepatocellular carcinoma [60,61] and that constitutes an excellent model for assessing the efficiency of gene delivery [62]. Although most evidence have shown that the PEI-GA_0.75_ and PEI-GL1_0.62_ conjugates were highly efficient in transfecting reporter genes in vitro and in vivo, most non-viral gene delivery studies continue to use reporter genes, warranting the need for additional studies by using an appropriate anti-oncogene to assess their hepatocellular carcinoma inhibition efficiency. We will also adopt strategies such as coating negatively charged polymers [63] or liposomes [38,56,61] at the above polyplexes to form ternary complex or lipo-polyplex, to further improve their transfection efficiency for in vivo use in our next study.

## 3. Materials and Methods

### 3.1. Materials and Cell Culture

Materials. PEI (1.8 K), glycyrrhizinic acid, glycyrrhetinic acid, ethylamine, Fluorescein isothiocyanate isomer I were purchased from Aladdin Inc. (Shanghai, China). Histamine, PEI (25 K) and 3- (4,5- two -2 -2,5- two phenyl methyl thiazole tetrazolium bromide salt) MTT, chlorpromazine, Filipin Ⅲ, wortmannin, cytochalasin D were purchased from Sigma-Aldrich (Shanghai, China). The pGL4.13 luciferase plasmid and luciferase assay kits were purchased from Promega (Madison, WI, USA). The EGFP and Trail plasmids was kindly provided by the Shanghai Institute of Materia Medica Chinese Academy of Sciences. The plasmids were amplified in Escherichia coli DH5α and extracted using Endo-Free Plasmid Kit (Qiagen, Hilden, Germany). The fetal bovine serum (FBS), antibiotics (penicillin 100 U/mL and streptomycin 100 μg/mL), DMEM medium and RPMI-1640 medium were obtained from GIBCO. The LabelIT^®^ Nucleic Acid Labeling Kit, and Cy^TM5^ were purchased from Mirus Bio (Madison, WI, USA). The Lyso Tracker Green and Hoechst 33342 were purchased from Invitrogen (Molecular Probes, Carlsbad, CA, USA).

Cell culture. The HepG2 cells (human hepatocellular carcinoma), SW480 (human colorectal adenocarcinoma) and A549 cells (human lung carcinoma) were purchased from the American Type Culture Collection and were cultured in DMEM (for HepG2 cells) or RPMI 1640 medium (for A549 and SW480 cells) with 10% FBS and 1% penicillin–streptomycin solution (complete medium). All cells were cultured in humidified atmosphere with 5% CO_2_ at 37 °C.

### 3.2. Preparation and Characterization of PEI-GA and PEI-GL Polyplexes

GA or GL modified PEIs were performed via N-acylation (Scheme 1) at room temperature. Taking PEI-GA_0.75_ for example, GA (0.235 g, 0.5 mmol) was conjugated to PEI (1800 Da, 0.9 g, 0.5 mmol) in the presence of EDC/NHS (N-(3-Dimethylaminopropyl)-N′-ethylcarbodiimide hydrochloride/N-Hydroxysuccinimide, 1.25-fold excess). The crude product of GA-PEI conjugates was dialyzed using a dialysis bag with a molecular weight cut-off of 1000 Da in DMSO for 48 h and then in deionized water for another 24 h, filtrated through 0.45 μm and finally lyophilized (Scheme S1 for detailed information). For GL modified PEIs, GL (0.412 g, 0.5 mmol) was firstly reacted with ethylamine solution in DMSO (0.092g, 1.03 mmol) or histamine solution (0.228 g, 1.03 mmol) for 24 h in the presence of NHS (0.144 g, 1.25 mmol) and EDC (0.24 g, 1.25 mmol). Then GL1 or GL2 solution was added dropwise into the PEI solution (1800 Da, 0.5 mmol, in DMSO) in the presence of EDC/NHS (1.5-fold excess) and stirred for another 24 h. Following the processing steps, the PEI-GL1- or PEI-GL2 crude products were treated with the same method that was used for PEI-GA and as mentioned above. All the PEI−GA and PEI-GL conjugates were characterized by 1HNMR (400 MHz, Bruker, Karlsruhe, Germany, D_2_O) and UV absorbance (Molecular Device Spectra Max M2e, San Jose, CA, USA), and Fourier-transform infrared spectrometer (FT-IR) (Spectrum 100, Platinum Elmer, Waltham, MA, USA).

1HNMR (400 MHz, Bruker, Germany, D_2_O). PEI-GA_0.75_: δ: 5.5 (1H, s, CH=), 3.4-2.2 (243, broad, NHCH_2_CH_2_), 2.0-0.43 (43H, broad). PEI-GA_1.1_, δ: 5.5 (1.21H, s CH=), 3.4-2.2 (143H, broad, NHCH_2_CH_2_), 2.0-0.43 (43H, broad). PEI-GA_1.7_, δ: 5.5 (1H, s, CH=), 3.4-2.2 (87.3H, broad, NHCH_2_CH_2_), 2.0- 0.43 (43H, broad). PEI-GL1_0.62_, δ: 5.5(1.6H, s, H=), 3.7-2.2 (218.8, broad, NHCH_2_CH_2_), 2.0-0.43 (50H, broad). PEI-GL2_0.65_, δ: 7.6 (1.65H, s, Imidazole ring), δ: 6.7 (1.62H, s, Imidazole ring), δ: 5.5 (0.81H, s, CH=), 3.7-2.2 (211.5H, broad, NHCH_2_CH_2_), 2.0-0.43 (44H, broad).

All the PEI-GA and PEI-GL polyplexes were freshly prepared before characterization or gene transfection experiments. Generally, each quantified PEI-GA or PEI-GL conjugates solution (at various weight ratios, in 10 mM HEPES) was added into 40 μg/mL (100 μg/mL for in vivo transfection) plasmid pDNA (luciferase plasmid or enhanced green fluorescent protein (EGFP)) solution. Then each sample was incubated for 30 min at room temperature. The positive control, PEI1.8K and PEI25K polyplexes were prepared at their optimal weight ratio [38,56,64].

The sizes and zeta potentials of polyplexes were carried out by a Malvern Zetasizer Nano 3600 apparatus (Malvern, UK) at 25 ℃. The morphologies of PEI-GA_0.75_/pDNA and PEI-GL1_0.62_/pDNA polyplexes at weight ratio of 3, were acquired by transmission scanning electron microscope (TEM, Hitachi-H7000, Tokyo, Japan) after staining with phosphato-tungstic acid (PTA).

### 3.3. Gel Retardation Assay

The pDNA binding capacity and heparin competitive binding assay of PEI-GA and PEI-GL conjugates were investigated by agarose gel (Bio-Rad, CA, USA) electrophoresis. All the conjugates/pDNA polyplexes at weight ratios from 0.25 to 6 were electrophoresed on a 1% (*w*/*v*) agarose gel at 100 V for 20 min and visualized under a UV illumination (CLINX, Shanghai, China). For pDNA release assay, 0.2–1 U heparin vital units were added to freshly prepared polyplexes at the weight ratio of 2 or 3, and then the mixed solutions were incubated for another 30 min, and finally analyzed by agarose gel electrophoresis as described above.

### 3.4. Cytotoxicity of PEI-GA and PEI-GL Conjugates

The cytotoxicity of PEI-GA and PEI-GL conjugates was examined in HepG2, A549 and SW480 cells by MTT assay. The cells were seeded in 96-well plates at a density of approximatively 6000 cells per well and cultured for 24 h in 100 μL DMEM or RPMI-1640 containing 10% FBS. The cells were treated with conjugates at different concentrations for 48 h. Then 20 μL of stock solution of MTT (5 mg/mL in PBS) was added to each well and incubated for 3 h until dark blue formazan crystals were visible. After centrifuging at 2800 rpm for 5min, the medium was discarded and 100 μL of DMSO was added to dissolve the formazan crystal. The absorbance at 562 nm and 620 nm were recorded using a microplate spectrophotometer. The cell viability (%) was calculated relative to the control and according to the following equation: Cell viability% = (OD_562_–OD_620_) _conjugates_/(OD_562_–OD_620_) _control_ × 100%.

### 3.5. Evaluation of Transfection Efficiency in Vitro

HepG2, SW480 and A549 cells (3.0 × 10^4^ cells/well) were seeded in 48-well plates with 400 μL of DMEM or RPMI-1640 complete medium. When the cells reached for 70–80% confluence, the medium was replaced with 400 μL of serum-free medium. Then, 50 μL of PEI-GA/pDNA or PEI-GL/pDNA polyplexes at weight ratios of 1.5, 3, 4.5 and 6 was added and incubated with cells for 4 h. Then, the complete medium was replaced, and the cells were cultured for an additional 44 h. The complete medium was discarded and the cells were rinsed with 500 μL PBS. Finally, after thorough lysis of the cells with 100μL lysate buffer (1X), 5μL of luciferin (Promega, Madison, WI, USA) was added to 20 μL of cell lysate in each tube, and luciferase gene expression was quantified by photon counting with a luminometer (Berthold FB12, Pforzheim, Germany) [38,56]. The protein concentration was measured using a Bradford assay kit (Sangon biotech, Shanghai, China). The value of relative luciferase light units per milligram protein was obtained by the measured RLU value divided by the protein concentration. All data are presented as the mean of at least three independent measurements and each measurement was performed in triplicate.

To observe EGFP (expressing green fluorescence protein) and TRAIL expression, cells were seeded onto glass-bottom dishes at a density of 1.5 × 10^5^ cells per dish in 1.5 mL of 10% FBS-containing cell culture medium and incubated at least 24 h. The medium was replaced with 1.5 mL of fresh serum-free medium and a certain amount of polyplex solutions. The EGFP transfection procedure was similar to that of the above luciferase transfection procedure. The green fluorescence was observed by a confocal laser scanning microscope (CLSM, Nikon-A1 system, Tokyo, Japan) and quantitatively analyzed by flow cytometry (BD FACS CaliburTM, New Jersey, USA).

### 3.6. Free GA Competition Assay

A free GA competition assay was conducted in order to determine the receptor mediated cellular entry of the GA or GL modified PEIs. HepG2 and SW480 cells were seeded in 48-well plates at a density of 3 × 10^4^ cells per well and incubated for 24 h prior to transfection. The culture medium was replaced with serum-free medium and different certain amounts of GA (0, 1, 2, 3 μg) were added. After incubation for 30 min, 50 μL of PEI-GA_0.75_, PEI-GL1_0.62_ and PEI-GL2_0.65_ polyplexes (1 μg pLUCI/well) at their optimal weight ratios, were added to each well. The remaining transfection procedure was the same as the above.

### 3.7. Endocytosis Inhibitor Effects on Cellular Uptake and Transfection

Endocytosis inhibitors were used to identify the potential involvement of the endocytosis pathways [51,54,65]. The DNA was labeled with Cy5 according to the manufacturer’s instructions. HepG2 cells were seeded in 12-well plates at a density of 1.5 × 10^5^ cells per well in 1 mL of medium and incubated for 24 h. The medium was replaced with 1 mL of fresh serum-free medium. Chlorpromazine, Filipin Ⅲ, Genistein, Wortmannin or Cytochalasin D were respectively added to the serum-free medium at the concentrations of 50, 7.5, 200, 5 or 5 μM at 37 °C or 4 °C for 30 min. Then 15 μL of cy5 labeled pDNA polyplex solution was added to each well and incubated for 2 h. Again then, the medium was discarded and the cells were rinsed with 2 mL of PBS. Subsequently the cells were treated with 0.25% trypsin/EDTA for 30 s, and collected by centrifugation, and resuspended in 0.4 mL of PBS. The harvested cells were immediately analyzed by flow cytometry to determine cy5-positive cell percentage (a total of 10,000 cells were counted per treatment).

The effect of each inhibitor on luciferase gene transfection was performed according to the above transfection procedure and to the inhibitors’ pretreatment protocol.

### 3.8. Observation of Intracellular Trafficking by Confocal Imaging

HepG2 cells were seeded onto glass-bottom dishes at a density of 1.5 × 10^5^ cells per dish in 1.5 mL of medium and incubated for at least 24 h. The cells were incubated with cy5 labeled polyplexes for 0.5 h, 2 h and 5 h according to the above transfection protocol. The cells were incubated with Lyso Tracker Green (green), at a concentration of 200 nM for 1 h, to label the lysosomes, and with Hoechst 33342 (blue) at a concentration of one drop/mL medium for 20 min, to stain the nucleus before observation. Finally, the medium was discarded and the cells were washed thrice with PBS before observation by a confocal laser scanning microscope.

### 3.9. Transfection in Vivo

The animal studies were approved by the Animal Care and Use Committee of Zhejiang University and were performed in strict accordance with the NIH guidelines for the care and use of laboratory animals (NIH Publication No. 85-23 Rev. 1985). Female BALB/c athymic mice (6–8 weeks old) at an average weight of 20.0 ± 2 g, were obtained from SLAC Laboratory Animal Co. Ltd. (Shanghai, China). The animals were housed in sterile cages within laminar airflow hoods in a specific pathogen-free room with a 12-h light/12-h dark schedule and fed autoclaved chow and water ad libitum. The HepG2 intraperitoneal tumor model was established via i.p. inoculation of 4 × 10^6^ HepG2 cells in 0.2 mL of PBS into the right flank belly cavities of nude mouse using a previously reported method used in our laboratory [38,61].

For luciferase gene transfection in vivo, mice were randomly grouped (*n* = 4). Twenty days after the inoculation, the PEI-GA_0.75_/pLUCI and PEI-GL1_0.62_/pLUCI solutions (0.2 mL) at a dose of 0.5 mg pLUCI/Kg were i.p. injected. The PEI 1.8 K polyplexes was used as a positive control. After 48 h transfection, the mice were i.p. injected with D-luciferin sodium salt (Gold Biotechnology, St. Louis, USA) at dose of 150 mg/kg body weight in 0.2 mL PBS. Five minutes later, the mice were imaged under anesthesia with 2.5% isofluorane using a Xenogen IVIS Lumina system (Caliper Life Sciences, Boston, USA) (2-min exposure per image). The acquired images were obtained by superimposing the emitted light over the grayscale photographs of the animals. The quantitative analysis was performed using the Lumina II Living Image 4.2 software (Boston, MA, USA).

For the quantitation of tissue luciferase expression, the mice were sacrificed. Forty-eight hours after transfection and tumor tissues were dissected and lysed with lysis buffer (Promega, Madison, WI, USA). The in vivo luciferase gene expression was quantified as the transfection protocol in vitro. All data were presented as the mean of at least 4 independent measurements.

### 3.10. Statistical Analysis

All data were expressed as the mean ± the standard error of the mean. All statistical analyses were performed using GraphPad Prism 7 software (GraphPad Software, Inc, La Jolla, CA, USA). The differences between 2 groups were analyzed by paired t test. *p* values < 0.05 was regarded as statistically significant.

## 4. Conclusions

In this study, we developed a couple of new artificial virus gene vectors by directly coupling hydrophobic glycyrrhetinic acid (GA) and glycyrrhetinic acid (GL) to PEI1.8K. These novel liver-targeting conjugates had several unique features: (I) They were readily prepared by one or two steps of N-acetylation reaction. (Ⅱ)All the polyplexes had small sizes (< 80 nm) under their optimum weight ratio. Additionally, the low substituted conjugates (feed ratio 1:1) revealed superior pDNA condensing ability. (Ⅲ) PEI-GA_0.75_, PEI-GL1_0.62_ and PEI-GL2_0.65_ conjugates exhibited low toxicity, high selectivity and efficient gene expression on HepG2 cells in vitro and in vivo. (Ⅳ) Attributed to GA-receptor mediated endocytosis and energy-independent (caveolae- and clathrin-independent) transduction, PEI-GA_0.75_ and PEI-GL1_0.62_ showed excellent specificity to HepG2 cells. (Ⅴ) Compared with GA modified PEIs, GL modified PEIs achieved better transfection efficiency and lower toxicity due to the better balance of hydrophobicity/hydrophilicity, which was needed to gain the desired polyplex properties for an efficient gene delivery. This study provides a new perspective for the design of efficient and specific liver-targeting non-viral gene vectors.

## Figures and Tables

**Table 1:** Amount substitution of PEI-GA and PEI-GL.

Item	Feed Ratio	Substitution Amount		Yield
		(by OD)	(by 1HNMR)	
PEI:GA	1:1	0.75	0.69	67.8%
	1:2	1.10	1.16	54.1%
	1:3	1.71	1.89	45.8%
PEI:GL1	1:1	0.62	0.66	51.6%
PEI:GL2	1:1	0.65	0.67	52.8%

## Supplementary Materials

Supplementary materials can be found at https://www.mdpi.com/1422-0067/20/20/5074/s1.
