# Supplementary material for: Glycyrrhizin Acid and Glycyrrhetinic Acid Modified Polyethyleneimine for Targeted DNA Delivery to Hepatocellular Carcinoma
Source: Int J Mol Sci. 2019 Oct 12;20(20):5074. doi: 10.3390/ijms20205074 (PMC6829341; doi:10.3390/ijms20205074)
Supplement: Supplementary file 1 [file ijms-20-05074-s001.zip › Supporting informatio1.docx]

**Supporting information**

Glycyrrhizin acid and Glycyrrhetinic acid Modified Polyethyleneimine for Targeted DNA Delivery to Hepatocellular Carcinoma

Mingzhuo Cao ^1,2*^, Yong Gao ^3^, Mengling Zhan^2^, Nasha Qiu ^1^, Ying Piao^1^, Zhuxian Zhou^1^, Youqing Shen ^1^

^1^ Center for Bio-nanoengineering and Key Laboratory of Biomass Chemical Engineering, Ministry of Education, College of Chemical and Biological Engineering, Zhejiang University, Hangzhou 310027, China;

^2^ Scientific Research and Experimental Center, Henan University of Chinese Medicine, Zhengzhou, 450058, China.

^3^ Henan province food and drug Administration, Food and Drug Evaluation and Inspection Center, Zhengzhou, 450018, China.

***** Corresponding Author: Mingzhuo Cao, E-mail: [cmz8199@126.com](mailto:cmz8199@126.com)

***Synthesis of PEI- GA, PEI-GL and FITC-PEI-GA, FITC-PEI-GL1, conjugates.***


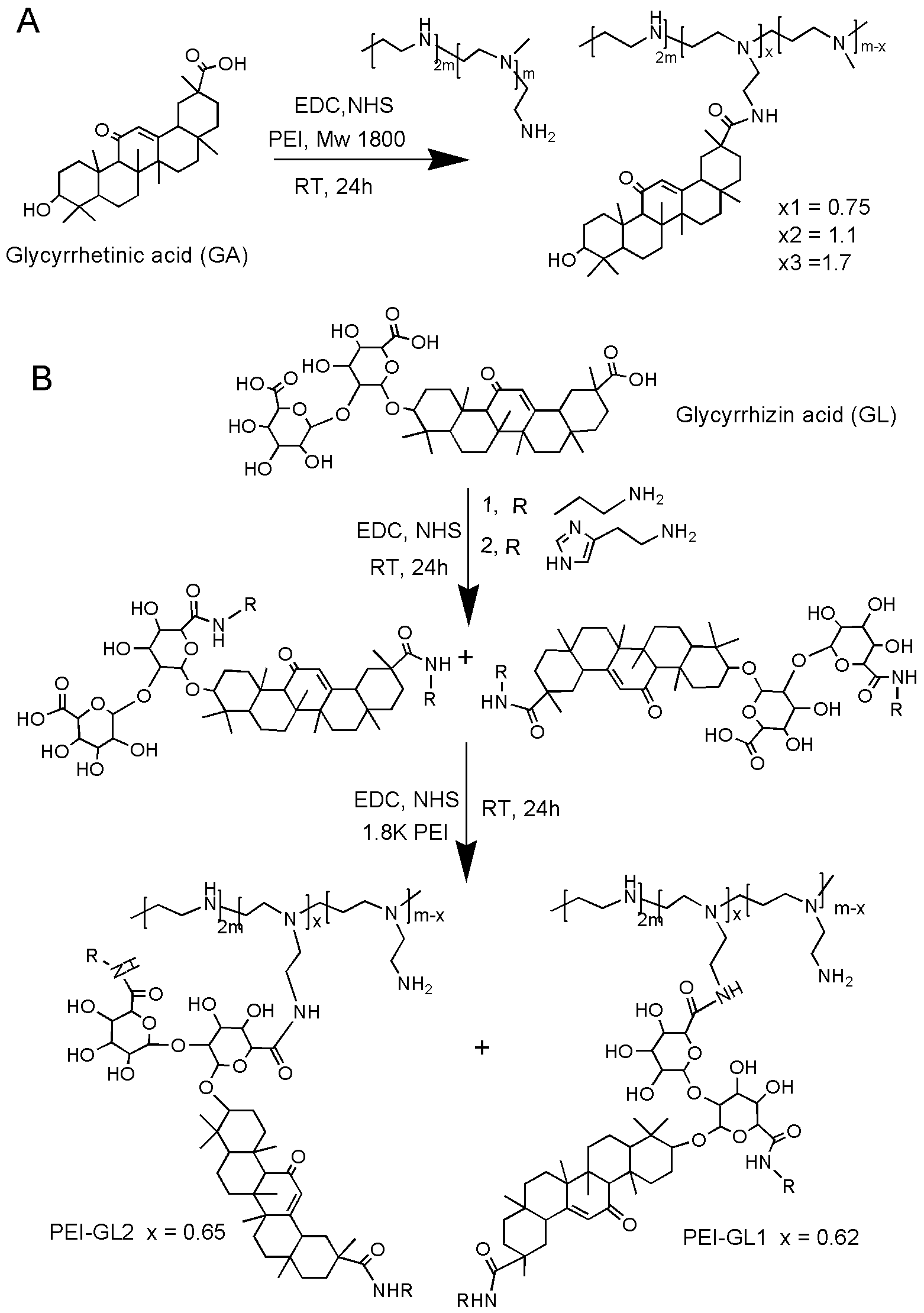


Scheme S1. Synthetic routes of PEI-GA (A) and PEI-GL (B).

Substitution amount of GA or GL were calculated from 1HNMR (400 MHz, Bruker, Germany, D_2_O). And detailed information about the characterization of the conjugates is given as follows: PEI-GA_0.75_: δ: 5.5(1H, s, CH=), 3.4 -2.2 (243, broad, NHCH_2_CH_2_), 2.0- 0.43 (43H，broad). PEI-GA_1.1_, δ: 5.5(1.21H, s CH=), 3.4 -2.2 (143H, broad, NHCH_2_CH_2_), 2.0- 0.43 (43H, broad). PEI-GA_1.7_, δ: 5.5(1H, s, CH=), 3.4 -2.2 (87.3H, broad, NHCH_2_CH_2_), 2.0- 0.43 (43H, broad). PEI-GL1_0.62_, δ: 5.5(1.6H, s, H=), 3.7-2.2 (218.8, broad, NHCH_2_CH_2_), 2.0- 0.43 (50H, broad). PEI-GL2_0.65_, δ:7.6(1.65H, s, Imidazole ring), δ: 6.7(1.62H, s, Imidazole ring), δ: 5.5(0.81H, s, CH=), 3.7-2.2(211.5H, broad, NHCH_2_CH_2_), 2.0-0.43 (44H, broad).

***Synthesis of FICT-PEI- GA and FICT-PEI-GL1 conjugates.***

Synthesis of fluorescent dye-labeled PEI-GA_0.75_ and PEI-GL1_0.62_ 100mg PEI-GA_0.75_ or PEI-GL1_0.62_ and fluorescein isothiocyanate (FITC) (0.8 mg) were dissolved in 6mL deionized water or DMSO. And the reaction mixtures were stirred overnight at room temperature and dialyzed in deionized water to remove the free dye molecules for 24h and finally lyophilized to obtain ginger powders.

**In intro transfection efficiency.**

Just like PEI25K, the presence of 10% FBS led to a significant reduction in gene expression and similar results were observed in all modified PEIs polyplexes, while the inhibition effects were much attenuated.


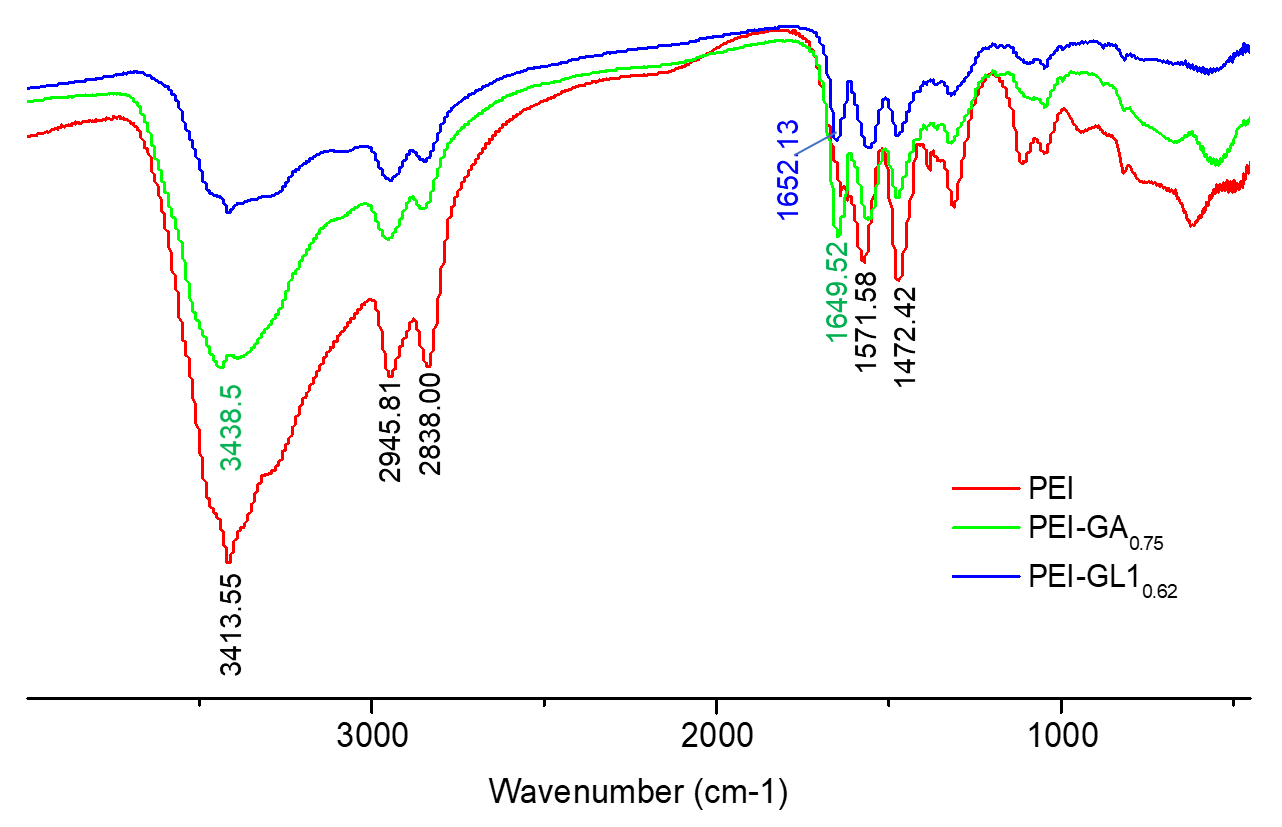


Figure S1. IR characterization of PEI-GA_0.75_, PEI-GL1_0.62_ and PEI.


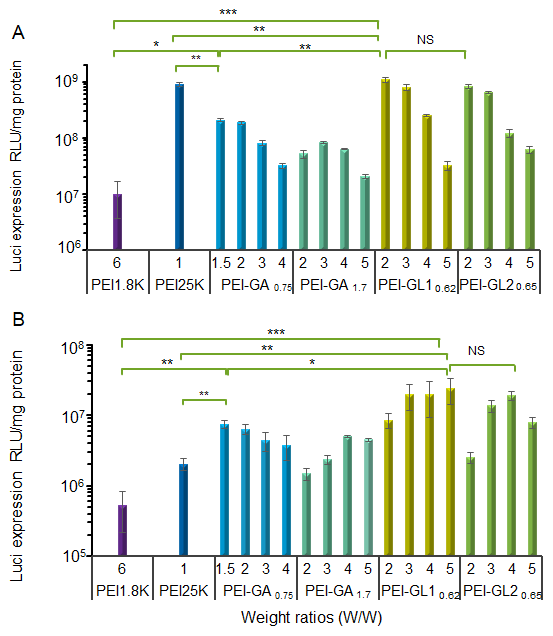


**Figure S2.** In vitro gene transfection efficiency. Luciferase expression efficiency of PEI-GA/pLUCI and PEI-GL/pLUCI polyplexes and the controls (PEI.8K and PEI25K) in serum-free medium on A549 cancer cells with 2.25μg/ml Luciferase plasmid concentration. Results are shown as mean ± SD (n = 3). Statistically significant differences are indicated by *p < 0.05, **p < 0.01 and ***p < 0.001 using t-test (Unpaired).


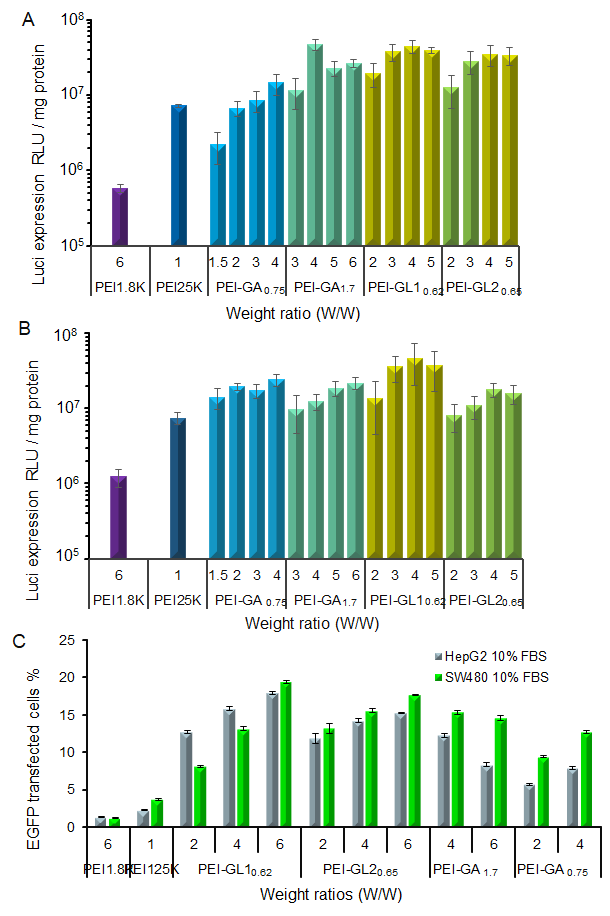


Figure S3. In vitro gene transfection efficiency of PEI-GA and PEI-GL conjugates and the controls under 10% FBS. Luciferase expression efficiency on HepG2 (A) and SW480 (B) cancer cells with 2.25μg/ml Luciferase gene concentration. Results are shown as mean ± SD (n = 3). (C) GFP expression efficiency of the four polyplexes and the controls on SW480 and HepG2 cells with 2.5μg/mL EGFP gene concentration. And GFP-positive cells measured by ﬂow cytometry.

Figure S4. In vitro TRAIL expression induced killing of SW480 cancer cells. Images of cells after being treated with PEI-GL1_0.62_ /pTRAIL polyplexes (w/w 3) and PEI/pTRAIL polyplexes (PEI25K, w/w 1) at various DNA doses for 4 h in serum-free medium (A) or containing 10% FBS medium (B), and then cultured in fresh medium for 44 h. All the scale bars represent 10 μm.


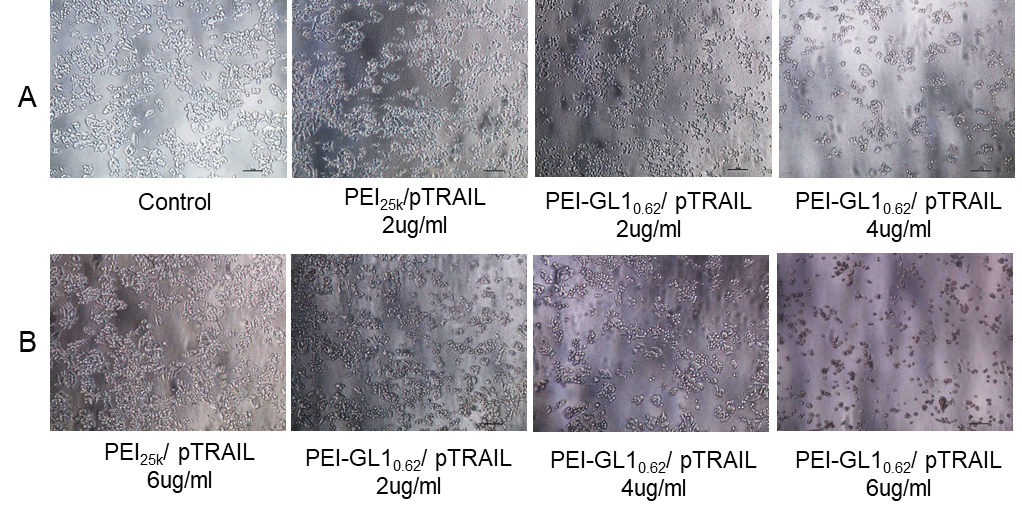


**PEI-GA_1.1_ conjugate transfection efficiency in vitro.**


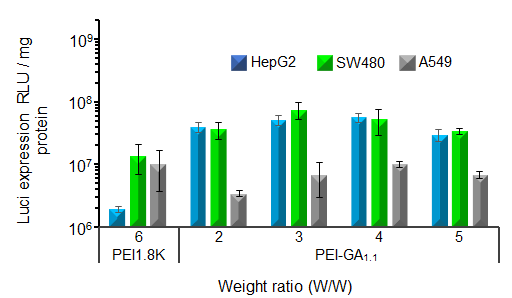


Figure S5. In vitro gene transfection efficiency of PEI-GA_1.1_ conjugate and PEI 1.8K in serum-free medium on HepG2 cells, SW480 cells and A549 cancer cells with 2.25μg/ml Luciferase plasmid concentration. Results are shown as mean ± SD (n = 3).

**Endocytosis inhibitor effects on cellular uptake and transfection**

Genistein was investigated to further illustrate intracellular trafficking of ^FITC-^PEI-GA_0.75_ and ^FITC-^PEI-GL1_0.62_ conjugates by using a combination of flow cytometry and laser confocal microscope. As shown in Figure S5 A and B, flow cytometry results showed that cellular uptake of the two polyplexes were suppressed by the genistein inhibitor, resulted in a 30.8% and 64.1% decrease in intracellular uptake. Laser confocal microscope result confirmed the cellular uptake inhibition (Figure 7C). Genistein was a tyrosine kinase inhibitor, known to disrupt caveolae-dependent endocytosis and caveolae & clathrin independent endocytosis [[1](#_ENREF_1), [2](#_ENREF_2)]. Caveolin pathway is an acknowledged beneficial pathway for gene transfection[[3](#_ENREF_3), [4](#_ENREF_4)], in which vectors were initially localized into caveosomes to avoid pDNA lysosomal degradation [[5](#_ENREF_5)]. Some studies reported that HepG2 cell have very poor expression of caveolin [[6](#_ENREF_6), [7](#_ENREF_7)], but another studies also confirmed that in liver and in different hepatocyte cell lines, the internalization of specific ligands was through caveolae, e.g. LDL receptor[[8](#_ENREF_8)]. It was a pity that there is no direct evidence to confirm GA receptor located in caveolae. Combining the endocytosis inhibitors results and the role of GA receptors, we can infer that HepG2 cells endocytose PEI-GA_0.75_ and PEI-GL1_0.62_ polyplexes through caveolae & clathrin independent pathway.


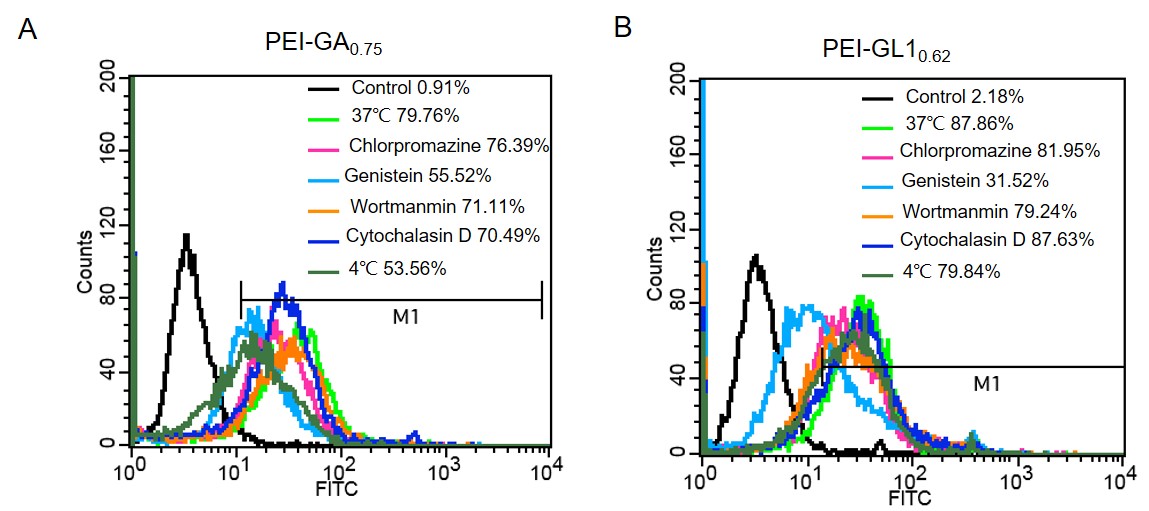


Figure S6. Endocytosis inhibitor effects on cellular uptake of ^FITC-^PEI-GA_0.75_/pDNA (A) and ^FITC-^PEI-GL1_0.62_/pDNA (B) polyplexes. HepG2 cells were pretreated with each inhibitor for 0.5 h and cultured with ^FITC-^PEI-GA_0.75_/pDNA (A) and ^FITC-^PEI-GL1_0.62_/pDNA (B) for 2h in serum-free medium, then measured by flow cytometry.

**Reference**

[1] Sahay G., Alakhova D. Y., Kabanov A. V. Endocytosis of nanomedicines. *J Control Release*, **2010**, *145*: 182-95.

[2] Duncan R., Richardson S. C. Endocytosis and intracellular trafficking as gateways for nanomedicine delivery: opportunities and challenges. *Mol Pharm*, **2012**, *9*: 2380-402.

[3] Katye M. Fichter Nilesh P. Ingle, Patrick M. McLendon, and Theresa M. Reineke Polymeric Nucleic Acid Vehicles Exploit Active Interorganelle Trafficking Mechanisms. *ACS NANo*, **2013**, *7*: 347-64.

[4] Huang H., Cao D., Qin L., et al. Dilution-stable PAMAM G1-grafted polyrotaxane supermolecules deliver gene into cells through a caveolae-dependent pathway. *Mol Pharm*, **2014**, *11*: 2323-33.

[5] Scholz C., Wagner E. Therapeutic plasmid DNA versus siRNA delivery: common and different tasks for synthetic carriers. *J Control Release*, **2012**, *161*: 554-65.

[6] Song W., Gregory D. A., Al-Janabi H., et al. Magnetic-silk/polyethyleneimine core-shell nanoparticles for targeted gene delivery into human breast cancer cells. *Int J Pharm*, **2019**, *555*: 322-36.

[7] Gabrielson N. P., Pack D. W. Efficient polyethylenimine-mediated gene delivery proceeds via a caveolar pathway in HeLa cells. *J Control Release*, **2009**, *136*: 54-61.

[8] Bourgeois To Quyen Truong， Dominique Aubin， Philippe. Opposite effect of caveolin-1 in the metabolism of high-density and low-density lipoproteins. *Biochimica et Biophysica Acta*, **2006**, *1761*: 24-36.
